# Lean Body Weight-Tailored Iodinated Contrast Injection in Obese Patient: Boer versus James Formula

**DOI:** 10.1155/2018/8521893

**Published:** 2018-08-13

**Authors:** Damiano Caruso, Domenico De Santis, Flaminia Rivosecchi, Marta Zerunian, Nicola Panvini, Marta Montesano, Tommaso Biondi, Davide Bellini, Marco Rengo, Andrea Laghi

**Affiliations:** ^1^Department of Radiological, Oncological and Pathological Sciences, “Sapienza” University of Rome, Sant'Andrea University Hospital, Via di Grottarossa 1035, Rome, Italy; ^2^Department of Radiological, Oncological and Pathological Sciences, “Sapienza” University of Rome, Via Franco Faggiana 1668, Latina, Italy

## Abstract

**Purpose:**

To prospectively compare the performance of James and Boer formula in contrast media (CM) administration, in terms of image quality and parenchymal enhancement in obese patients undergoing CT of the abdomen.

**Materials and Methods:**

Fifty-five patients with a body mass index (BMI) greater than 35 kg/m^2^ were prospectively included in the study. All patients underwent 64-row CT examination and were randomly divided in two groups: 26 patients in Group A and 29 patients in Group B. The amount of injected CM was computed according to the patient's lean body weight (LBW), estimated using either Boer formula (Group A) or James formula (Group B). Patient's characteristics, CM volume, contrast-to-noise ratio (CNR) of liver, aorta and portal vein, and liver contrast enhancement index (CEI) were compared between the two groups. For subjective image analysis readers were asked to rate the enhancement of liver, kidneys, and pancreas based on a 5-point Likert scale.

**Results:**

Liver CNR, aortic CNR, and portal vein CNR showed no significant difference between Group A and Group B (all *P* ≥ 0.177). Group A provided significantly higher CEI compared to Group B (*P* = 0.007). Group A and Group B returned comparable overall subjective enhancement values (3.54 and vs 3.20, all *P* ≥ 0.199).

**Conclusions:**

Boer formula should be the method of choice for LBW estimation in obese patients, leading to an accurate CM amount calculation and an optimal liver contrast enhancement in CT.

## 1. Introduction

Contrast media (CM) enhancement, during CT exams, is influenced by multiple factors: patients and tissue characteristics, CM type, volume, and concentration, injection time, and scan timing [1–5]. The easiest CM injection protocol implemented in clinical practice consists of injecting a fixed amount of CM [6] or tailoring the CM to patient weight [1, 4, 7].

The concentration of CM in parenchymal organs is closely related to extracellular fluid volume space and plasma [8]. In obese subjects, a large proportion of body weight consists of adipose tissue, which is poorly perfused compared to solid parenchymas and in which CM distributes poorly [9]. Consequently, the adipose tissue does not substantially contribute to contrast distribution. Therefore, such approach can result in an over- or underdosage of CM in the obese population [10].

Boer [11] and Peters et al. [12] reported that lean body weight (LBW) better correlates with extracellular fluid volume rather than total body weight (TBW). This parameter has been demonstrated to better estimate the optimal CM dose calculation compared to TBW, reducing also patient-to-patient variability [13, 14]. However, LBW is not currently calculated with consistency among investigators. In fact, several formulas [11, 13, 15–17] have been used to serve this purpose.

While James formula [15] is one of the most widely applied formulas in clinical practice [18], despite general agreements in the advantage of using LBW over TBW to assess the right amount of CM, major concerns have been raised regarding the optimal formulas to be applied in obese population. In fact, Nyman [19] proposed Boer formula [11] could better estimate LBW in obese patients.

Therefore, the aim of our study was to prospectively compare the performance of James and Boer formula in terms of image quality and parenchymal enhancement in obese patients.

## 2. Material and Methods

### 2.1. Study Population

This prospective, single-center, HIPAA study was approved by our Institutional Review Board. Written informed consent was obtained from all subjects included in our study.

From July 2016 to October 2017 patients referring at our institution for a multiphasic CT of the abdomen, including arterial, portal venous, and equilibrium phases, with a body mass index (BMI) ≥ 35 kg/m^2^, were enrolled in this study. Exclusion criteria were as follows: age < 18 years, previous reactions to iodinated intravenous CM, impaired renal function (estimated glomerular filtration rate < 30 mL/min/1.73m^2^) or any other contraindication to CM injection, intravenous access smaller than 18 gauge, and pregnancy. Patient sex, age, height, weight, body mass index (BMI), and LBW were recorded.

### 2.2. Patient Randomization and Lean Body Weight Calculation

Patients were randomized into two groups with a 1:1 ratio. All the individuals allocated in Group A had the LBW calculated applying Boer formula [11], according to patient's sex, as follows:

(1) LBWmaleBoer=0.407·W+0.267·H−19.2

(2) LBWfemaleBoer=0.252·W+0.473·H−48.3

where* W* represents the patient weight in kilograms and* H* the patient height in meters.

All the individuals allocated in Group B had the LBW calculated applying James formula [15] as follows:

(3) LBWmaleJames=1.10·W−128·W2100·H2

(4) LBWfemaleJames=1.07·W−148·W2100·H2

where* W* represents the patient weight in kilograms and* H* the patient height in meters.

### 2.3. CT Image Acquisition

All examinations were performed using a 64-row multidetector CT scanner (Lightspeed VCT, GE Medical Systems, Waukesha, WI, USA). All acquisitions were performed with the patient in supine position, in craniocaudal direction, with a z-axis coverage from the diaphragmatic dome to the pubic symphysis. Scanning parameters were adjusted as follows: tube voltage, 120 kVp; beam pitch, 1.375:1; detector configuration, 64 · 0.625 mm. A z-axis tube current modulation (Smart mA, GE Healthcare) was applied with a noise index of 28 (min/max tube current: 200/600 mAs) as recommended by the manufacturer for abdominal CT.

Contrast media (Iobitridol 350, Guerbet, France) was intravenously administered through an 18-gauge antecubital intravenous access using an automated dual-syringe power injector (Stellant D; Medrad Inc, Warrendale, PA) at a flux of 4.5 mL/s followed by a 50 mL saline flush at the same flow rate. Each patient received 0.7 g of iodine [gI] per kg of LBW. The resulting value was subsequently divided by CM concentration to obtain the exact CM volume to be injected, as follows:

(5) $$\begin{array}{c} {CM}_{volume\left(ml\right)}=\frac{0.7∙LBW}{350}∙1000 \end{array}$$

Scan timing was determined by a 120 kVp bolus tracking technique (SmartPrep, GE Healthcare) by placing a region-of-interest (ROI) in the abdominal aorta at the level of the celiac tripod. The threshold for scan initiation was set at an attenuation of100 Hounsfield Units (HU). A triphasic acquisition protocol was applied as follows: the late arterial phase was acquired 18s after reaching the threshold, while the portal venous phase and the equilibrium were acquired 70s and 180s after reaching the threshold, respectively. For the specific purpose of this study only the portal venous phase was analyzed.

### 2.4. CT Image Reconstruction

Image datasets were reconstructed at the CT scanner console with the following parameters: section thickness of 1.25 mm and spacing of 1.25 mm. Iterative reconstruction (ASiR; GE Healthcare) was applied at strength level of 40%, as recommended by the vendor.

### 2.5. Objective Image Quality Analysis

All datasets were transferred to an independent workstation (Advantage workstation 4.5, GE Healthcare). A reader with 10 years of experience in abdominal imaging performed the image analysis in axial sections. HU was measured by placing a circular ROI of approximately 1 cm^2^ (mean pixel number: 600; range: 200-1200) in the liver (n = 3 ROIs; segment II, IVa, and VII), suprarenal abdominal aorta, portal vein, and left psoas muscle. An additional ROI was placed in the subcutaneous fat and standard deviation (SD) was calculated and defined as image noise. To ensure data robustness, all measurements were repeated 3 times and subsequently averaged. Liver HU was defined as the mean of the 3 averaged ROIs.

Liver, aorta, and portal vein contrast-to-noise ratio (CNR) was calculated as follows:

(6) CNRliver=HUliver−HUmuscleNoiseCNRaorta=HUaorta−HUmuscleNoiseCNRportal  vein=HUportal  vein−HUmuscleNoise

The liver contrast enhancement was quantified by calculating the contrast enhancement index (CEI) as follows [18]:

(7) $$\begin{array}{c} CEI={HU}_{enhanced}-{HU}_{unenhanced} \end{array}$$

### 2.6. Subjective Image Quality Analysis

Subjective analysis was performed by two radiologists in consensus with three and seven years of experience in abdominal CT imaging, respectively. Readers were blinded to the formula used to calculate the LBW. The datasets were initially displayed at a preset window width of 400 HU and window level of 40 HU; however, readers were allowed to manually adjust window width and level settings according to their preferences.

Readers were asked to rate the enhancement of liver, kidneys, and pancreas by means of a 5-point Likert scale [20]: 1 = very poor; 2 = poor; 3 = fair; 4 = good; 5 = excellent.

### 2.7. Statistical Analysis

All statistical analyses were performed by using the MedCalc^®^ Satistical Software version 17.9.7 (MedCalc Software bvba, Ostend, Belgium; http://www.medcalc.org; 2017). Variables are expressed as mean ± SD. The Kolmogorov-Smirnov test was used to assess data distribution. Patient characteristics, CNRs, CEI, and subjective image quality, were compared between the two groups. In case of normally distributed data, Student's* t*-test was applied. In case of nonnormally distributed data, Wilcoxon signed-rank test was used. Statistical significance was defined as a two-tailed* P* value < 0.05.

## 3. Results

### 3.1. Study Population

From an initial population of 68 individuals, 13 patients were excluded from the study due to an inadequate intravenous access. Thus, the final population consisted of 55 patients. Patients underwent CT for oncologic follow-up (n = 41), suspected cancer (n = 4), cirrhosis (n = 3), abdominal pain (n = 3), hepatic haemangiomas (n = 1), echinococcal cyst (n = 1), unexplained persistent fever (n = 1), and urinary infection (n = 1).

There were no significant differences in patient characteristics between the two groups. Patient characteristics are reported in Table 1.

### 3.2. Contrast Media Dose

Group A received 43.28 ± 7.07 gI, corresponding to 123.65 ± 20.19 mL of CM.

Group B received 41.93 ± 8.32 gI, corresponding to 119.79 ± 23.79 mL of CM. No statistical differences were observed between the two groups (*P* = 0.532) as shown in Table 1.

### 3.3. Objective Image Quality

Comprehensive objective image quality data are reported in Table 2.

**Figure 1:**
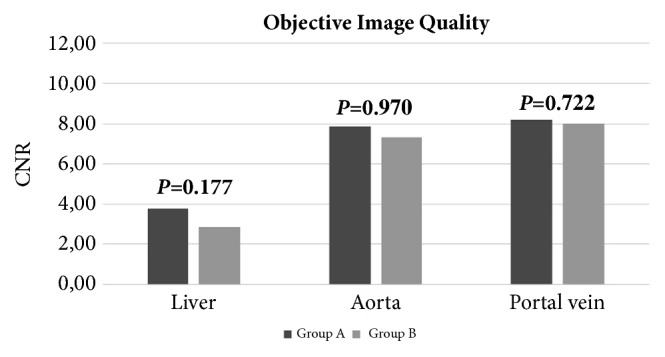
Graph bars of CNR of liver, aorta, and portal vein for groups A and B. All the differences between the two groups were not significant.

Group A achieved slightly higher liver CNR (3.75 ± 2.31 vs. 2.83 ± 2.65), aortic CNR (8.15 ± 4.19 vs. 7.95 ± 3.99), and portal vein CNR (7.82 ± 7.24 vs. 7.27 ± 3.76); however, none of the values reached statistical significance (all *P* ≥ 0.177) as shown in Figure 1.

**Figure 2:**
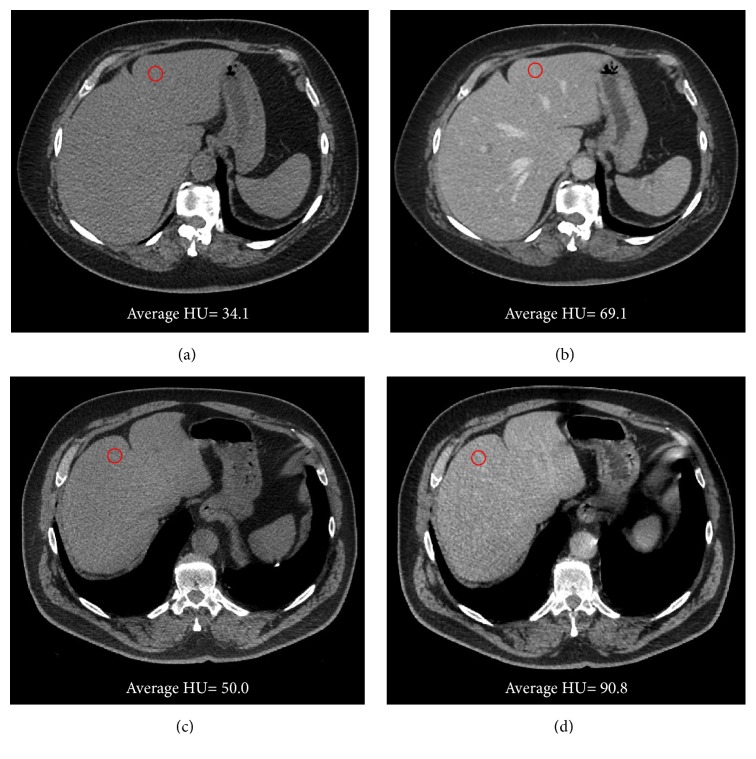
Axial CT scan of unenhanced (a) and portal venous phase (b) of a 63-year-old female (LBW: 49.1 kg; CM volume: 98 mL) allocated in Group A and unenhanced (c) and portal venous phase (d) of a 73-year-old female (LBW: 47.7 kg; CM volume: 95 mL) allocated in Group B. The patient in Group A achieved a higher CEI (62.0 HU) compared to the patient in Group B (40.8 HU). Windows settings: width: 400; level: 40.

Group A provided significantly higher CEI (51.45 ± 9.79) compared to Group B (41.79 ± 14.32, *P* = 0.007), Figure 2.

### 3.4. Subjective Image Quality

**Figure 3:**
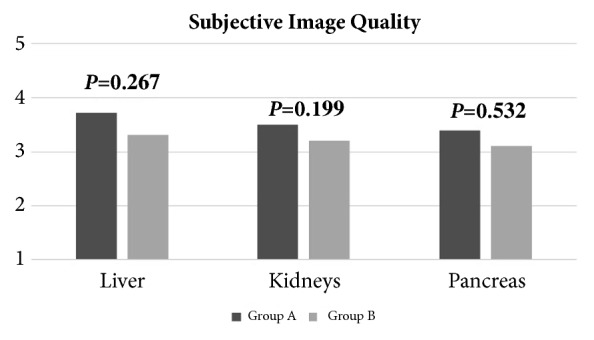
Subjective image analysis results. Liver, kidneys, and pancreas enhancement was ranked on a 5-point Likert scale. Graph bars of subjective image quality scores achieved by liver, kidneys, and pancreas in the two groups. All the differences were not significant.

Detailed results are displayed in Figure 3. Group A and Group B returned comparable overall enhancement values (3.54 and vs 3.20, all *P* ≥ 0.199). In Group A, two patients (7.7%) scored poor contrast enhancement while 1 patient (3.8%) achieved very poor contrast enhancement. In Group B, three patients (10.3%) achieved poor contrast enhancement and other three patients (10.3%) scored a very poor contrast enhancement.

## 4. Discussion

The aim of our study was to compare, on CT of the abdomen, the performance of James and Boer formula in terms of image quality and parenchymal enhancement in obese patients. CNR of liver, aorta, and portal vein and liver CEI and image quality have been analyzed on portal venous phase. Our results demonstrate both formulas achieve comparable image quality, while Boer formula allowed for higher enhancement compared to James formula.

A recently published multicentre study [9], performed on 1342 patients with a normal BMI, established LBW as the best parameter to determine the optimal amount of CM in CT examinations. Authors determined aortic and hepatic attenuation during unenhanced, hepatic arterial phase, and portal venous phase and, among different body size parameters, LBW exhibited the strongest correlation with aortic and hepatic enhancement. In the aforementioned investigation, LBW was estimated by James formula. However, Nyman [19] pointed out the limitations of such formula in assessing LBW in obese patients, proposing Boer formula as a reliable method do achieve a consistent LBW measurement in obese population due to its linear function. Results of our investigations did not find statistical significant differences in terms of CNR between the two formulas, despite the fact that our patient population had an average BMI slightly below 40 kg/m^2^. A possible explanation could be due to the fact that LBW calculation with James formula reaches a plateau at a BMI of about 37 and 43 kg/m^2^ in women and men, respectively. Therefore, despite being classified as* obese*, our study population was still the BMI threshold making James formula ineffective. Thus, this result is in accordance with Nyman's statement [19] and, at the same time, further strengthens the validity of James formula in calculating LBW for a large percentage of patients [10, 13, 21] and in other imaging modalities such as hybrid PET/CT [22]. Both Ho et al. [10] and Kondo et al. [13] investigations achieved optimal vascular and hepatic enhancement on portal venous phase and unenhanced and portal venous phase, respectively, administrating CM dose estimated on LBW calculated with James formula.

Interestingly, the implementation of Boer formula provided a significantly higher CEI compare to James formula. A CEI of at least 50 HU is advisable in clinical practice for adequate liver imaging and diagnostic purposes [4, 23]. Some hypotheses can be formulated to try to explain this result, such as Boer formula, which allows for a more appropriate estimation of LBW in obese patients, or the slightly higher amount of CM administered in Group A, which could have led to higher CEI. However, the first hypothesis is in discordance with the nonstatistical differences in terms of BMI and LBW in both groups (Table 1) while the nonsignificant higher amount of CM in Group A seems to contradict the second hypothesis, given that the explanation of these results is difficult to be determined and it is possible that other patient-related variables, such as cardiac output [4, 5, 13, 24], have played a role in providing this result.

Both Boer and James formula provided a fair-to-optimal contrast enhancement of liver, kidneys, and pancreas in the vast majority of patients. 11% of patients whose LBW was calculated by Boer formula reported a poor or very poor enhancement while this percentage almost doubled (21%) in the Group in which James formula was applied. These results are quite in accordance with the aforementioned objective image quality results and give strength to the assumption that Boer formula outperforms James formula either in terms of subjective or in terms of objective image quality, allowing for a more reliable image evaluation since it is well established that an adequate contrast enhancement is crucial for multiple clinical evaluation of the abdomen, especially when parenchymal lesions are suspected and a multiphasic CT examination is required [25, 26].

Our study has some limitations. First, this investigation was conducted on a small sample size population and further studies on a larger sample size are advisable to further confirm our findings. Second, this preliminary study was only focused on image quality and diagnostic performance was not assessed. Third, both female and male patients were included in Group A and Group B, despite the fact that the two formulas applied to calculate LBW also take into account patient sex as well; a subgroup analysis on sex-separated cohorts would be advisable in future investigations.

In conclusion, Boer formula should be the method of choice for LBW estimation in class II obese patients, leading to an accurate CM amount calculation and an optimal liver contrast enhancement.

## Figures and Tables

**Table 1:** Patient characteristics.

**Parameter**	**Group A**	**Group B**	***P* value**
Number of patients	26	29	-
Male-to-female ratio	11:15	16:13	-
Age, years	61.54 ± 11.06	59.31 ± 10.67	0.493
Weight, kg	109.54 ± 20.93	109.38 ± 15.01	0.989
Height, m	165.85 ± 7.67	166.03 ± 13.94	0.469
BMI, kg/m^2^	39.79 ± 6.55	38.75 ± 4.11	0.501
LBW, kg	62.30 ± 10.15	61.21 ± 11.07	0.705
CM volume, mL	123.65 ± 20.19	119.79 ± 23.79	0.532

Data are mean ± SD.

BMI = body mass index; LBW = lean body weight; CM = contrast media

**Table 2:** Quantitative image analysis.

	**Group A**	**Group B**	***P* value**
	CNR		
Liver	3.75 ± 2.31	2.83 ± 2.65	0.177
Aorta	8.15 ± 4.19	7.95 ± 3.99	0.722
Portal Vein	7.82 ± 7.24	7.27 ± 3.76	0.967
	CEI		
Liver (HU)	51.45 ± 9.79	41.79 ± 14.32	0.007

Data are mean ± SD

CEI = contrast enhancement index; CNR = contrast-to-noise ratio

## Data Availability

The data used to support the findings of this study are available from the corresponding author upon request.

## Abbreviations

BMI: Body mass index
LBW: Lean body weight
CT: Computed tomography
HU: Hounsfield units
CNR: Contrast-to-noise ratio
CM: Contrast media
CE: Contrast enhancement
TBW: Total body weight.

## References

[1] Yamashita Y., Komohara Y., Takahashi M. (2000). Abdominal helical CT: Evaluation of optimal doses of intravenous contrast material - A prospective randomized study. *Radiology*.

[2] Goshima S., Kanematsu M., Kondo H. (2006). MDCT of the Liver and Hypervascular Hepatocellular Carcinomas: Optimizing Scan Delays for Bolus-Tracking Techniques of Hepatic Arterial and Portal Venous Phases. *American Journal of Roentgenology*.

[3] Awai K., Takada K., Onishi H., Hori S. (2002). Aortic and hepatic enhancement and tumor-to-liver contrast: Analysis of the effect of different concentrations of contrast material at multi-detector row helical CT. *Radiology*.

[4] Heiken J. P., Brink J. A., McClennan B. L., Sagel S. S., Crowe T. M., Gaines M. V. (1995). Dynamic incremental CT: Effect of volume and concentration of contrast material and patient weight on hepatic enhancement. *Radiology*.

[5] Bae K. T., Heiken J. P., Brink J. A. (1998). Aortic and hepatic contrast medium enhancement at CT: Part II. Effect of reduced cardiac output in a porcine model. *Radiology*.

[6] Saini S. (2004). Multi-detector row CT: Principles and practice for abdominal applications. *Radiology*.

[7] Awai K., Hiraishi K., Hori S. (2004). Effect of Contrast Material Injection Duration and Rate on Aortic Peak Time and Peak Enhancement at Dynamic CT Involving Injection Protocol with Dose Tailored to Patient Weight. *Radiology*.

[8] Bae K. T. (2010). Intravenous contrast medium administration and scan timing at CT: considerations and approaches. *Radiology*.

[9] Awai K., Kanematsu M., Kim T. (2016). The optimal body size index with which to determine iodine dose for hepatic dynamic CT: A prospective multicenter study. *Radiology*.

[10] Ho L. M., Nelson R. C., DeLong D. M. (2007). Determining contrast medium dose and rate on basis of lean body weight: Does this strategy improve patient-to-patient uniformity of hepatic enhancement during multi-detector row CT?. *Radiology*.

[11] Boer P. (1984). Estimated lean body mass as an index for normalization of body fluid volumes in humans.. *American Journal of Physiology-Endocrinology and Metabolism*.

[12] Peters A. M., Snelling H. L. R., Glass D. M., Love S., Bird N. J. (2010). Estimated lean body mass is more appropriate than body surface area for scaling glomerular filtration rate and extracellular fluid volume. *Nephron Clinical Practice*.

[13] Kondo H., Kanematsu M., Goshima S. (2010). Body size indexes for optimizing iodine dose for aortic and hepatic enhancement at multidetector CT: Comparison of total body weight, lean body weight, and blood volume. *Radiology*.

[14] Kondo H., Kanematsu M., Goshima S. (2011). Aortic and hepatic enhancement at multidetector CT: Evaluation of optimal iodine dose determined by lean body weight. *European Journal of Radiology*.

[15] DHSS/MRC Group on Obesity Research, Waterlow J. C., James W. P. T. (1976). *Research on Obesity*.

[16] Janmahasatian S., Duffull S. B., Ash S., Ward L. C., Byrne N. M., Green B. (2005). Quantification of lean bodyweight. *Clinical Pharmacokinetics*.

[17] Hume R. (1966). Prediction of lean body mass from height and weight. *Journal of Clinical Pathology*.

[18] Rengo M., Bellini D., Businaro R. (2017). MDCT of the liver in obese patients: evaluation of a different method to optimize iodine dose. *Abdominal Radiology*.

[19] Nyman U. (2016). James Lean Body Weight Formula Is Not Appropriate for Determining CT Contrast Media Dose in Patients with High Body Mass Index. *Radiology*.

[20] Setty B. N., Sahani D. V., Ouellette-Piazzo K., Hahn P. F., Shepard J.-A. O. (2006). Comparison of enhancement, image quality, cost, and adverse reactions using 2 different contrast medium concentrations for routine chest CT on 16-slice MDCT. *Journal of Computer Assisted Tomography*.

[21] Kidoh M., Nakaura T., Oda S. (2013). Contrast enhancement during hepatic computed tomography: Effect of total body weight, height, body mass index, blood volume, lean body weight, and body surface area. *Journal of Computer Assisted Tomography*.

[22] Tahari A. K., Chien D., Azadi J. R., Wahl R. L. (2014). Optimum lean body formulation for correction of standardized uptake value in PET imaging. *Journal of Nuclear Medicine*.

[23] Brink J. A., Heiken J. P., Forman H. P., Sagel S. S., Molina P. L., Brown P. C. (1995). Hepatic spiral CT: Reduction of dose of intravenous contrast material. *Radiology*.

[24] Rengo M., Bellini D., De Cecco C. N. (2011). The optimal contrast media policy in CT of the liver. Part I: Technical notes. *Acta Radiologica*.

[25] Oliva M. R., Saini S. (2004). Liver cancer imaging: Role of CT, MRI, US and PET. *Cancer Imaging*.

[26] Dahlman P., Semenas E., Brekkan E., Bergman A., Magnusson A. (2016). Detection and characterisation of renal lesions by multiphasic helical CT. *Acta Radiologica*.

